# Determination of crown discoloration and fluorescence induced by different medications used in regenerative endodontic procedures: An *ex vivo* study

**DOI:** 10.4317/jced.58114

**Published:** 2021-08-01

**Authors:** Pablo Betancourt, Cristina Bucchi, Sebastiana Arroyo-Bote

**Affiliations:** 1 Research Centre for Dental Sciences (CICO), Department of Integral Adult Dentistry, Universidad de La Frontera, Temuco, Chile; 2 Associate Professor at the Faculty of Medicine and Health Sciences. University of Barcelona. Spain. Coordinating Professor of ADEMA. University of the Balearic Islands. Spain. IDIBELL Researcher

## Abstract

**Background:**

Crown discoloration is an undesirable side effect of the use of triple antibiotic paste (TAP) in regenerative endodontic procedures (REPs). The aim of this ex vivo study was to assess the potential for tooth discoloration and induction of fluorescence associated with the use of TAP containing either doxycycline (DOX) or clindamycin (CLIN), and of calcium hydroxide (Ca(OH)2), by spectrophotometric analysis and confocal laser scanning microscopy (CLSM).

**Material and Methods:**

A total of forty single-rooted human teeth extracted by therapeutic indication were used. The root canals were enlarged using the step-back technique up to a K #80 file and were randomly divided into four experimental groups (10 specimens each): i)Ca(OH)2 group, ii)TAP with DOX group, iii) TAP with CLIN group, iv) Control group (no treatment). To quantify the change of color of the different groups of teeth included, the Vita Easyshade advance 4.0 spectrophotometer was used. CLSM was used to determine fluorescence.

**Results:**

After 28 days of action inside the root canal, no extreme discoloration was visible, to the naked eye, in any of the teeth included in the study. Under the conditions of this ex vivo study, TAP with DOX induced the highest crown discoloration among the medicaments tested. In contrast, Ca(OH)2 and TAP with CLIN did not induce crown discoloration after 28 days. The TAP with DOX group presented the highest fluorescence measurements.

**Conclusions:**

Considering the discoloration potential and fluorescence changes in TAP with DOX or other tetracyclines, and the cytotoxic effect of TAPs, we recommend the use of Ca(OH)2 for REPs.

** Key words:**Discoloration, doxycycline, clinadamycin, calcium hydroxide, endodontics.

## Introduction

Regenerative endodontic procedures (REPs) have been proposed as an alternative to apexification and conventional endodontic therapy for the treatment of immature permanent teeth with pulp necrosis. The goal of REPs is to regenerate the pulp-dentin complex, in order to allow continued root development including increased wall thickness and root apex maturation (1,2).

Disinfection of the root canal system is a crucial stage in tooth revitalization (3). Anastomoses, isthmic, lateral canals and apical deltas make the root canal system a morphologically complex network, thus appropriate release and penetration of antiseptic solutions into the root canal system is necessary to decrease the bacterial load (4).

Many REPs protocols have proposed the use of calcium hydroxide (Ca(OH)2), formocresol and antibiotic pastes to control endodontic infection (5). In recent years, several investigators have used antibiotic paste for the effective elimination of endodontic pathogens (6). Nowadays, triple antibiotic paste (TAP) is thought to be the most widely used intracanal antibiotic combination in REPs (6). The components of TAP are ciprofloxacin, metronidazole and minocycline (MINO) (1). The increased popularity of TAP is explained by its excellent antibacterial properties, both on the dentinal surface and inside the dentinal tubules (1,7). Multiple studies have shown the antibacterial effect of TAP on infected root canals (8,9,10); however, crown discoloration is an undesirable side effect of the use of TAP. Responsibility for discoloration has been attributed to MINO, a derivative of tetracycline. It can form calcium ion complexes, causing many types of staining in teeth (6). Tooth discoloration begins when the antibiotic paste penetrates the dentinal tubules. It is important to note that the discoloration effect caused by MINO can begin 24 hours after contact with dentin (11).

It has been reported that 40% of teeth treated with TAP present crown discoloration (5). This high percentage represents an important clinical complication. Substantial crown discoloration, especially in anterior teeth, leads to aesthetic problems, generating a negative impact on the patient’s quality of life. In fact, the resolution of aesthetic problems is the leading motive for patient preferences, over other dental procedures (12).

The American Association of Endodontists (AAE) has established clinical considerations to minimize the risk of crown discoloration due to TAP. These guidelines suggest sealing the pulp chamber with a dentin adhesive agent, releasing the TAP into the root canal through a syringe, and placing antibiotic paste just below the cemento-enamel junction (CEJ). Replacing MINO with another antibiotic, such as clindamycin (CLIN), amoxicillin or cephalosporin, has been also suggested to minimize the risk of crown discoloration (13).

To the best of our knowledge, the addition of CLIN to TAP has received little attention. REPs is a topic in endodontics in full development and has attracted the attention of clinicians and endodontic societies. It is therefore necessary to test new mixtures of compounds and evaluate their impact on tooth structure, such as discoloration.

Due to the high percentage of discoloured teeth associated with the use of TAP in REPs, the objective of this *ex vivo* study was to assess the potential for tooth discoloration and induction of fluorescence associated with the use of the intracanal medication commonly used in endodontics (TAP with DOX, TAP with CLIN and Ca(OH)2) by spectrophotometric analysis and confocal laser scanning microscopy (CLSM).

## Material and Methods

The study protocol was approved by the Clinical Research and Ethics Committee of the University of Barcelona (No. 2016–23). A total of forty single-rooted human teeth extracted by therapeutic indication were used. They were disinfected by immersion in 0.5% chloramine-T solution (Merck, Germany) for 48 h and subsequently stored in saline solution until use. Teeth were collected by random sampling, ensuring that they had no signs of radectomy or resorption, carious lesions, calcifications of the canalicular system, dilacerations greater than 30º, or signs of previous endodontic intervention. The samples were cleaned using Endo Pro Ultra zirconia Satelec® ultrasound tips (Dentsply Maillefer®, Ballaigues, Switzerland) and a 7/8 curette, Gracey (Hu-Friedy ®, Chicago, USA), to remove calcified tissue and periodontal ligament debris from the root surface.

-Root canal treatment

**Figure 1:**
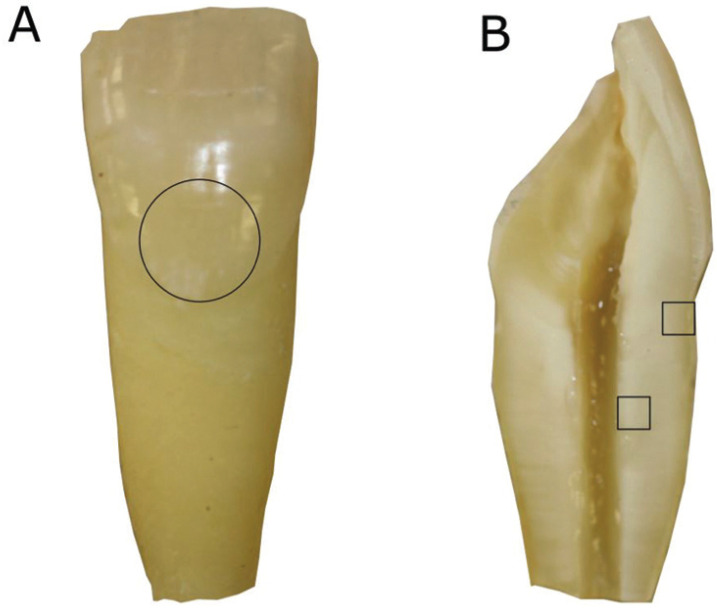
Maxillary incisor with canal instrumentation up to file #80 and with the apical third sectioned to simulate an immature tooth (A). Circle: Area of colour measurements with the spectrophotometer (A). After 28 days teeth were sectioned longitudinally and analysed by CLSM (B). Squares: areas of fluorescence measurement (B).

To simulate an immature tooth, the root apical part of each specimen was sectioned to obtain a standard root length of 10 mm below the labial CEJ (Fig. 1). The endodontic access cavity was performed with a high speed #10 diamond bur under water-cooling (Komet, Rock Hill, SC). The cervical third was prepared with Gates-Glidden burs. Apical permeability confirmation was checked with a K-File #10 (Dentsply Maillefer, Ballaigues, Switzerland). The root canals were enlarged using the step-back technique up to a K #80 file (Dentsply Maillefer, Switzerland). After the use of each file, the roots canals were irrigated with 2.5mL of 2.5% sodium hypochlorite (NaOCl), using a 30-gauge side-vented needle (Becton Dickinson, Madrid, Spain). The disinfection protocol ended with the irrigation of 1mL of 17% ethylenediaminetetraacetic acid (EDTA) for 3 minutes followed by 3 mL of saline. The forty single-rooted teeth were randomly divided into four experimental groups (10 specimens each) and treated according to the following protocols.

Group 1: Ca(OH)2 group. 40 mg Ca(OH)2 + 40mL serum.

Group 2: TAP with DOX group. Equal proportions of ciprofloxacin, metronidazole and DOX (20 mg each) + 2.4 mg methyl cellulose. The TAP components were mixed with sterile water to a final concentration of 0.1 mg / mL.

Group 3: TAP with CLIN group. Equal proportions of ciprofloxacin, metronidazole and CLIN (20 mg each) + 2.4 mg methyl cellulose. The TAP components were mixed with sterile water to a final concentration of 0.1 mg / mL.

Group 4: Control group (no treatment).

The apical foramen was covered with a layer of Parafilm (Heathrow Scientific, Vernon Hills, IL, USA). The antibiotic pastes (at final concentration of 0.1 mg / mL) and Ca(OH)2 were introduced into the root canals using a syringe. The root canals were filled with the medication to just below the labial CEJ and were refilled every week with their respective medication. The excess antibiotic paste in the access cavity was carefully removed with cotton pellets, then the access cavity was temporarily sealed with a cotton pellet to prevent possible colour change caused by temporary filling material. The specimens were inserted in hard silicone up to the CEJ and placed in a large petri dish with water, to prevent drying and to simulate the humid environment of the oral cavity. All samples were incubated at 37°C for 28 days.

-Colour measurement

**Figure 2:**
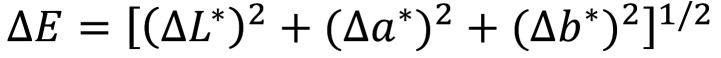
Formula.

To quantify the colour of the different groups of teeth included in the study, the Vita Easyshade advance 4.0 spectrophotometer (VITA, Germany) was used. The spectrophotometer defines three aspects of tooth colour through a CIELAB system (L*a*b*) and provides the following information: L*: brightness-darkness and value ranges from 0 (black) to 100 (white); a*: green (negative values) – red (positive values) value and b*: blue (negative values) – yellow (positive values). The data were substituted in the following formula to calculate the colour change (ΔE)14: (Fig. 2).

Colour measurements were taken on the buccal surface of the tooth, immediately above the CEJ (Fig. 1A). This ensured that the area analysed was the same for all the teeth. All measurements were recorded every seven days for the 28 days of the experiment. The colour was determined by one examiner after intra-calibration in a darkened room. The spectrophotometer was calibrated according to the manufacturer’s instructions before measuring colour shades.

-Fluorescence

**Figure 3:**
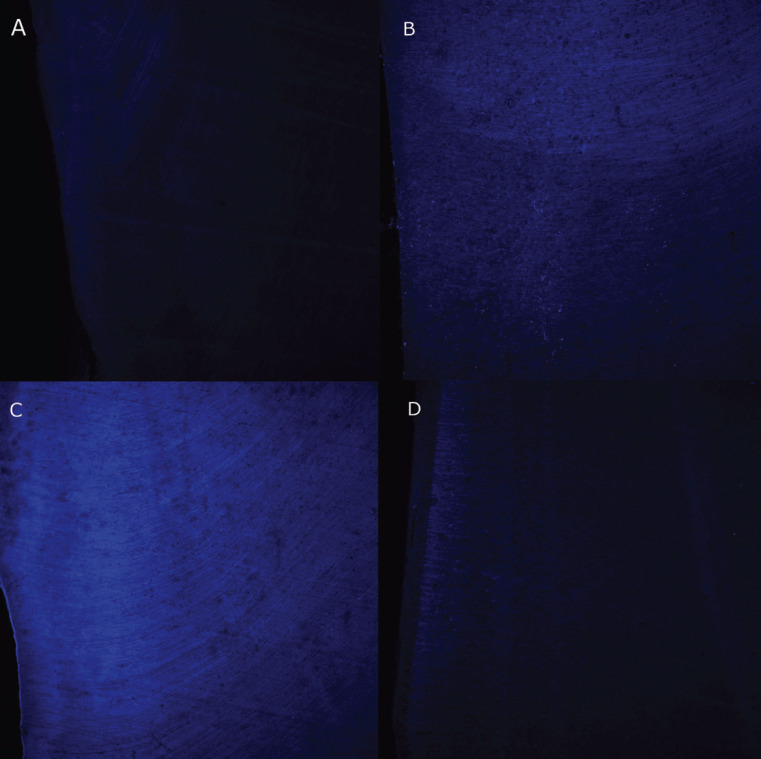
Representative CLMS images of teeth in the control (A), TAP with clindamycin (B), TAP with doxycycline (C), and calcium hydroxide (D) groups, in the inner middle third of the root canal.

At the end of the follow-up period, the teeth were sectioned along their longitudinal axis. The tooth halves were dried and mounted on glass slides and examined with a Zeiss LSM 880 spectral CLSM (Carl Zeiss, Jena, Germany) equipped with a 488-nm argon laser and 561-nm diode lasers. The zoom images were scanned at 40x by zoom immersion oil lens (1.3 numerical aperture), in an image format at a resolution of 1024x1024 pixels (Fig. 3). The CLSM images of the external aspect of the coronal third of the root, and of the internal aspect of the middle third (Fig. 1B) were obtained using ImageJ software (National Institutes of Health, Bethesda, MD, USA) and IMARIS software (Bitplane AG, Zurich, Switzerland). Fluorescence was quantified using the ImageJ Software

-Statistical Analysis

Statistical analyses were performed using SPSS 23.0 software (SPSS Inc, Chicago, IL). ANOVA (analysis of variance) of independent samples was used to compare multiple means and for post-hoc analysis. The TUKEY test was used with a significance of 5%.

## Results

**Figure 4:**
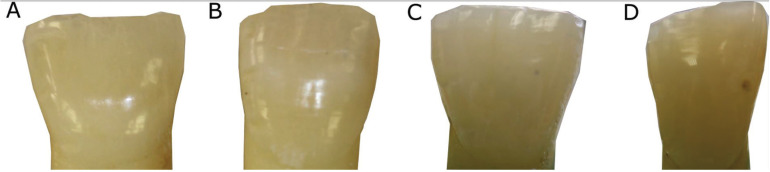
Representative images of control teeth (A), teeth treated with calcium hydroxide (B), TAP with Clindamycin (C) and TAP Doxycycline (D) after 28 days.

**Figure 5:**
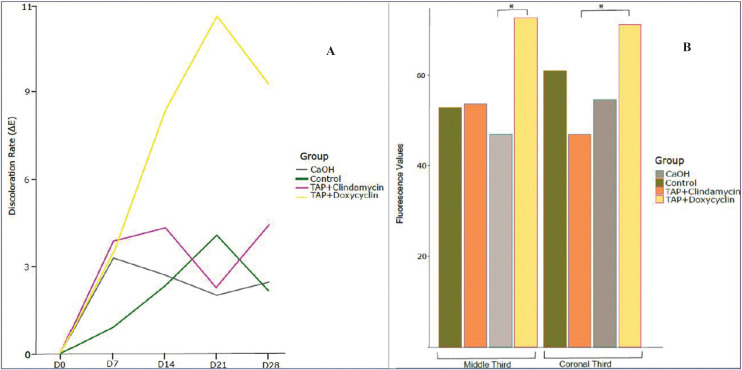
A: Discoloration rate (ΔE) of the different groups at days 0, 7, 14, 21 and 28, using Vita Easyshade spectrophotometer. B: Fluorescence values of the different groups in the middle and coronal thirds, after 28 days. *statistically significant difference (*P*<0.05).

After 28 days of action inside the root canal, to the naked eye, no extreme discoloration was observed in any of the teeth included in the study (Fig. 4). The colour changes measured by the Vita Esayshade spectrophotometer are shown in Table 1. The values obtained showed a certain degree of intrinsic discoloration of the tooth; the highest value was obtained for the TAP with DOX group (Fig. 5A), although the statistical analysis showed no significant differences among the groups (*p*> 0.05).

**Table 1:**
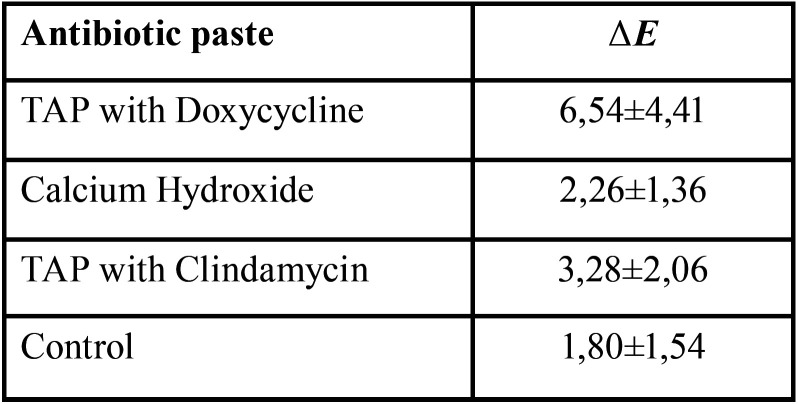
Mean values and standard deviation of ΔE with various antibiotic pastes after 28 days, 28, using Vita Easyshade spectrophotometer.

The results of fluorescence by CLSM are shown in Table 2. The TAP with DOX group obtained the highest fluorescence values in the middle and coronal thirds (Fig. 3C), showing statistically significant differences from the Ca(OH)2 group in the middle third (*p*= 0.016) and from the TAP with CLIN group in the apical third (*p*= 0.041).

**Table 2:**
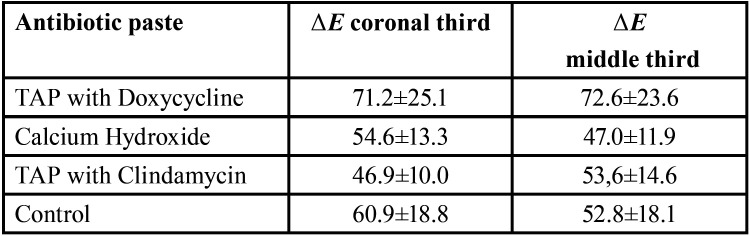
Mean values and standard deviation of ΔE with various antibiotic pastes by CLSM images.

## Discussion

Tooth discoloration has been reported in between 40% and 62% of REPs (5,15). This percentage is not negligible, considering that REPs has become the treatment of choice in immature permanent teeth with pulp necrosis. The discoloration reported after REPs can be attributed to the pulp necrosis itself, to the contamination of the dentinal tubule with blood after blood clot induction (16), to the MTA used as part of the protocol (15) and to the TAP used as intracanal medicament. This study analyzed the discoloration and fluorescence induced by medicaments commonly used in regenerative endodontics.

Several studies have shown that an intrinsic chemical modification is generated in the dentin structure on contact with TAP, which is observed as a colour change over time (11,17). TAP-induced discoloration is associated with chelation of calcium ions by the action of MINO (18). Despite this adverse side effect, TAP is still the most widely used intracanal medication in REPs because it has a broad spectrum of action on common endodontic pathogens, i.e., strict anaerobes and gram negative bacteria (19), and it has the ability to kill bacteria within the dentinal tubules (1).

Previous studies have suggested modifying the composition of TAP to prevent tooth discoloration, replacing MINO with another antibiotic, such as amoxicillin, cephalosporin or DOX (2,20-22). In the present study, the potential for tooth discoloration of DOX, CLIN and Ca(OH)2 were compared. The colour changes were evaluated objectively using a colorimeter. The spectrophotometric analysis showed that Ca(OH)2 and TAP with CLIN had similar values to the control group; TAP with DOX showed the highest discoloration values. Although DOX has been reported to show a significant reduction in the discoloration of the dental structure of extracted teeth when compared to the action of MINO (23), previous studies (24) observed that the application of TAP with DOX had a negative aesthetic impact resulting in visible crown discoloration after 21 days. Caution should be exercised in indicating this product, since it is a derivative of tetracycline and could have a chelating action similar to MINO, causing long-term discoloration.

Due to its antimicrobial effectiveness against acute infections and flare-ups (25), CLIN has been used as an intracanal medication in combination with other agents with good results (26), comparable even with the antimicrobial effect of TAP (27,28); however, it lacks antiresorptive properties (29). The use of CLIN in REPs has gained attention since it has been shown to not cause tooth discoloration when used inside the root canal (30). Rafatjou *et al*. (28) have recently concluded that there are no limitations on the clinical use of the new mixture of CLIN, metronidazole and ciprofloxacin in REPs. In our study, the TAP with CLIN group did not cause a visible change in colour after 28 days, which makes CLIN an option to replace MINO in TAP formulation. In view of the results reported in this article, CLIN may be considered as a potential substitute for MINO in antibiotic paste mixtures. However, previous studies of REPs have shown adverse side effects of antibiotics on stem cell survival, with less than 20% viability (31); and that CLIN could demineralize dentin. It may also degrade superficial collagen (32), affecting adhesion of stem cells to dentin and consequently their differentiation into an odontoblast-like phenotype. The application of antibiotic pastes in the root canal should therefore be limited.

The time of application of the antibiotic paste in the root canal has been a matter of discussion. The revascularization technique introduced by Banch and Trope (33) recommended an application time of 4 weeks. However, over the years, the time of use of the medication has varied between 1 and 4 weeks in different experimental studies (5). In the current investigation, we treated the root canal for 4 weeks, following the recommendations of the AAE (13).

Observation by CLSM allowed us to determine the distribution patterns of medication pastes in the dentin of the treated samples by fluorescence. More marked fluorescence was identified in the TAP with DOX group, as has been observed also in some case reports (34,35). The fluorescence values in the Ca(OH)2, TAP with CLIN and control groups were similar, showing that these medicaments had no effect on tooth fluorescence.

Traditionally, Ca(OH)2 has been used in apexification procedures. Its use has some disadvantages since long-term exposure seems to increase root fracture risks. However, the application of Ca(OH)2 shows favourable results in revascularization and root development of immature necrotic teeth after long-term follow-up (36,37). Ca(OH)2 has again attracted favourable attention in REPs because it has been suggested that it is a stem cell-friendly medication (31,38), and significantly improves the attachment of apical papilla cells to dentin (39). In agreement with our observations, Akay *et al*. (24) and Lenherr *et al*. (40) reported that Ca(OH)2 medication did not show crown discoloration at any of the time points measured.

The limitation of this study is that the experiments were made on extracted teeth. *Ex vivo* models cannot fully simulate clinical conditions. On the other hand, the methodology used in the present study has been validated in other articles (16,24). This is a first step in the development of a line of research, and further clinical studies are needed to evaluate the outcome of this protocol in REPs

Considering the discoloration potential and fluorescence changes in TAP with DOX or other tetracyclines, and the cytotoxic effect of TAPs, we recommend the use of Ca(OH)2 for REPS.

## Conclusions

Under the conditions of this *ex vivo* study, TAP with DOX induced the highest crown discoloration among the medicaments tested. In contrast, Ca(OH)2 and TAP with CLIN did not induce crown discoloration after 28 days. The TAP with DOX group presented the highest fluorescence measurements. The fluorescence values in the Ca(OH)2, TAP with CLIN and control groups were similar.
